# No hay paso: barriers to nature access faced by Mexicans in southern Arizona

**DOI:** 10.3389/fpubh.2025.1654101

**Published:** 2025-11-14

**Authors:** Rebecca M. Crocker, Josselyne Diomara Sánchez, Gabriel Alvin Cruz

**Affiliations:** 1 Health Promotion Sciences, University of Arizona, Tucson, AZ, United States; 2 Cruz Farm, Douglas, AZ, United States

**Keywords:** Mexican immigrants, nature barriers, NIMHD research framework, One Health, US–Mexico border, planetary health, migration-related stress, health benefits of nature

## Abstract

**Introduction:**

Mexican immigrants and other low-income populations in the United States face reduced access to natural environments, limiting their opportunities both to secure a wide range of associated health advantages and to participate fully in environmental stewardship and protection efforts. This ethnographic study was designed to investigate Mexicans’ access to and engagement with natural environments over the course of international migration from Mexico to southern Arizona to help fill important gaps in our understanding of the intersectional nature barriers faced in this population.

**Methods:**

We conducted interviews with stakeholders and historical experts (*n* = 9) and first- generation Mexican immigrants working in land-based careers (*n* = 10) to explore nature barriers in a current and historical context. Analysis was conducted utilizing a One Health adaptation of the National Institute of Minority Health and Health Disparities research framework.

**Results:**

Our analysis revealed barriers across all six levels of influence (planetary, interspecies, society, community, interpersonal, and individual) and multiple domains, including aridification of land, international migration, disruption to interspecies’ relationships, discrimination, lack of time and energy, and emotional distress tied to immigration status.

**Discussion:**

This article confirms multilevel barriers identified in the literature as well as highlights additional barriers not previously recorded. This suggests the need for further research and especially health interventions geared to increase immigrants’ access to nature to improve their health and heighten their ability to serve as effective advocates and stewards of the natural world.

## 1 Introduction

Immigrant and ethnic minority groups in the United States face reduced access to natural environments, limiting their opportunities both to secure a wide range of associated health advantages and to participate fully in environmental stewardship and protection efforts (1, 2). Known barriers to nature among Latino immigrants include limited knowledge about and poor perceived quality of local natural areas and parks in addition to lack of transportation and distance from open spaces (3, 4). In addition, immigrants have been shown to have limited time and energy for outdoor pursuits and to face language barriers and fears surrounding immigration surveillance (5, 6, 25).

Inadequate access to green spaces impedes nature’s health promoting effects and has even been shown to contribute to inequitable patterns in preventable deaths (7). The potential health benefits of nature time are vast, ranging from reducing obesity and cardiovascular and metabolic disorders to improving mental health and reducing the burden of loneliness and stress (8–11). Scientists have argued that our ties to the natural environments may be literally trapped in our genes, suggesting that biology rather than culture alone may explain many enduring facets of human-nature interdependence (12, 13). Such work, coupled with the theoretical contributions of Indigenous science, have bolstered the argument that natural environments are “healing spaces” and “therapeutic environments” that confer deeply seeded if oft intangible benefits to well-being and spiritual groundedness (14, 15).

While research suggests that green spaces act as an integral protective factor in natural cause mortality for all people (16), immigrants and others who face intensive periods of dislocation may stand to benefit more deeply from time spent in nature (17). Natural environments can enable a “re-emplacement” for migrants who suffer dramatic changes to locale. Studies among US based Latino immigrants suggest that time in green spaces aids in adapting to new host societies by easing stress, promoting social ties and integration into new environments, and stabilizing ties to home through activities like outdoor sports teams with co-nationals (4, 6, 18). This may be especially so for immigrants of Mexican origin, whose concepts of identity and psychological orientation are often “merged with the land” [(19), p. 189].

Yet we know little about immigrants’ barriers to nature access as compared to the barriers they face to other forms of health promotion, such as medical care and healthy built environments. Moreover, existing studies have focused primarily on urban regions in large immigrant dense states and tend to collapse diverse sub-populations, thereby blurring intra-group variations at the level of nativity and length of residence in the U. S. (20–22). Given these deficiencies in the research to date, immigrants’ knowledge about the character and implications of the barriers they face has not been fully explored, an act of epistemic injustice that further reinforces existing hierarchies around who knows the land and has the answers we need to manage and protect it (62). Delbaere et al. (23) have identified the need to employ more ethically grounded qualitative approaches to highlight diverse voices in the study of reciprocal human-environment relations.

This article seeks to help address these gaps by using historically and regionally grounded ethnographic methods to explore barriers to nature exposure among first-generation Mexican immigrants living in both rural and urban regions of southern Arizona. We apply a One Health lens to problematize nature exposure more systematically as a form of health promotion grounded in deep human ties to the natural world on a planetary level. Building off theories of human-nature intersectionality, a One Health approach forwards the argument that a complete study of human health —particularly among people facing displacement and other adverse conditions— must foreground people’s relationships with the natural world.

## 2 Materials and methods

### 2.1 Study aims

This ethnographic study was designed to investigate patterns in Mexicans’ access to and engagement with natural environments over the course of international migration from Mexico to southern Arizona. The lead author is a medical anthropologist who conducted prior ethnographic research among first generation Mexican immigrants in southern Arizona revealing interruptions to natural spaces during migration among people whose worldviews and healing frameworks were deeply influenced by ties to the natural world (5, 24–25).

### 2.2 Project design

The lead author developed a semi-structured interview guide for stakeholders working to promote nature access among Mexicans in southern Arizona and historical experts on Mexican history in the region, as well as a more in-depth interview guide for first generation immigrants. The latter contained sections on (1) childhood land-based experiences and practices, (2) the migration and displacement process, (3) the re-establishment of nature connections post-migration, and (4) links between nature access and health.

For the immigrant interviews, the first author utilized the “Go-Along Interview” in which participants host the researcher in trusted and familiar spaces according to their own schedules. This affords participants greater power in the research endeavor and enhances connectivity, empathy and understanding, which may be particularly important for research among immigrants and others facing societal disadvantage vis-a-vis language and other factors (26, 27). In addition, the Go-Along method elevates the use of environmental and spatial cues, which enhanced our ability to ground conversations with research participants in their natural environments (28).

### 2.3 Data collection

The lead author conducted the first set of interviews with stakeholders and historical experts (*n* = 9) from February–July of 2021. Initial participants were identified based on their record of scholarship or prior working relationship with the first author in academic or community contexts, and a snowball sample was utilized to recruit subsequent participants. Seven expert interviews were conducted by Zoom video calls, while the remaining two were conducted in-person. Interviews lasted between 55 and 100 min long and were audio recorded. The questions relevant to this article drawn from the historical background interviews included: (1) In what ways do you think Arizona has been inhospitable, foreign, or alien for Mexicans and Mexican origin people historically and in the present?, and (2) Have there been structural barriers that have limited Mexicans’ ability to establish ties to land in Arizona?

Following the initial review of interview transcripts, the first author finalized the immigrant interview guide and initiated the second phase of research. The first and second authors conducted interviews with first-generation Mexicans working in land-based careers (*n* = 10) between July 2021 and April 2022. Initial recruitment was conducted at a community garden as well as with an immigrant mutual aid society, and subsequently via a snowball sample. Six interviews were conducted on-site at community gardens, an agricultural heritage site, and ranches. These interviews included guided tours of the locations (on foot, by horseback, and in utility vehicles). Four participants chose to be interviewed in their homes or in a private location at the lead author’s University. Interviews lasted between 1 and 3.5 h (average 1.5 h) and were audio recorded. Questions eliciting information on barriers to nature access were woven through all four sections of the interview guide (see Table A1).

### 2.4 Data analysis

Audio files were uploaded into a secure, password protected University owned web platform and then transcribed in their original language by the second author, a bilingual and bicultural graduate student. Transcripts were uploaded into Dedoose qualitative coding software^1^.

This study employed inductive analysis to test the applicability of the One Health adaptation of the National Institute of Minority Health and Health Disparities (NIMHD) research framework with the given data set (29). The third author, a second-generation Mexican American farmer in southern Arizona with lived experience of facing challenges to accessing local resources, highlighted the importance of using a multi-faceted lens that would best represent the kaleidoscope of challenges that Mexicans face in accessing nature.

The NIMHD research framework was published in 2017 to promote health disparity research based upon a multi-dimensional approach attendant to the complexities outlined in the socioecological model in which human health is affected by risk and resilience at the individual, interpersonal, community, and societal levels (30, 31). This argument has been a critical step in evolving health science research beyond a simplified focus on health behaviors and genetic determinants of disease and toward a more complex understanding of the intersectionality of social and biological determinants of health (32, 33). The NIMHD research framework likewise builds off the National Institute on Aging model in which health determinants in the domains of biological, behavioral, built environment, and sociocultural environment may produce and shape health disparities (34). The NIMHD framework incorporates a life-course approach to capture the cumulative impacts of adverse events in early life, on-going exposure to social and environmental stressors, and intergenerational processes (30, 35).

**Figure 1:**
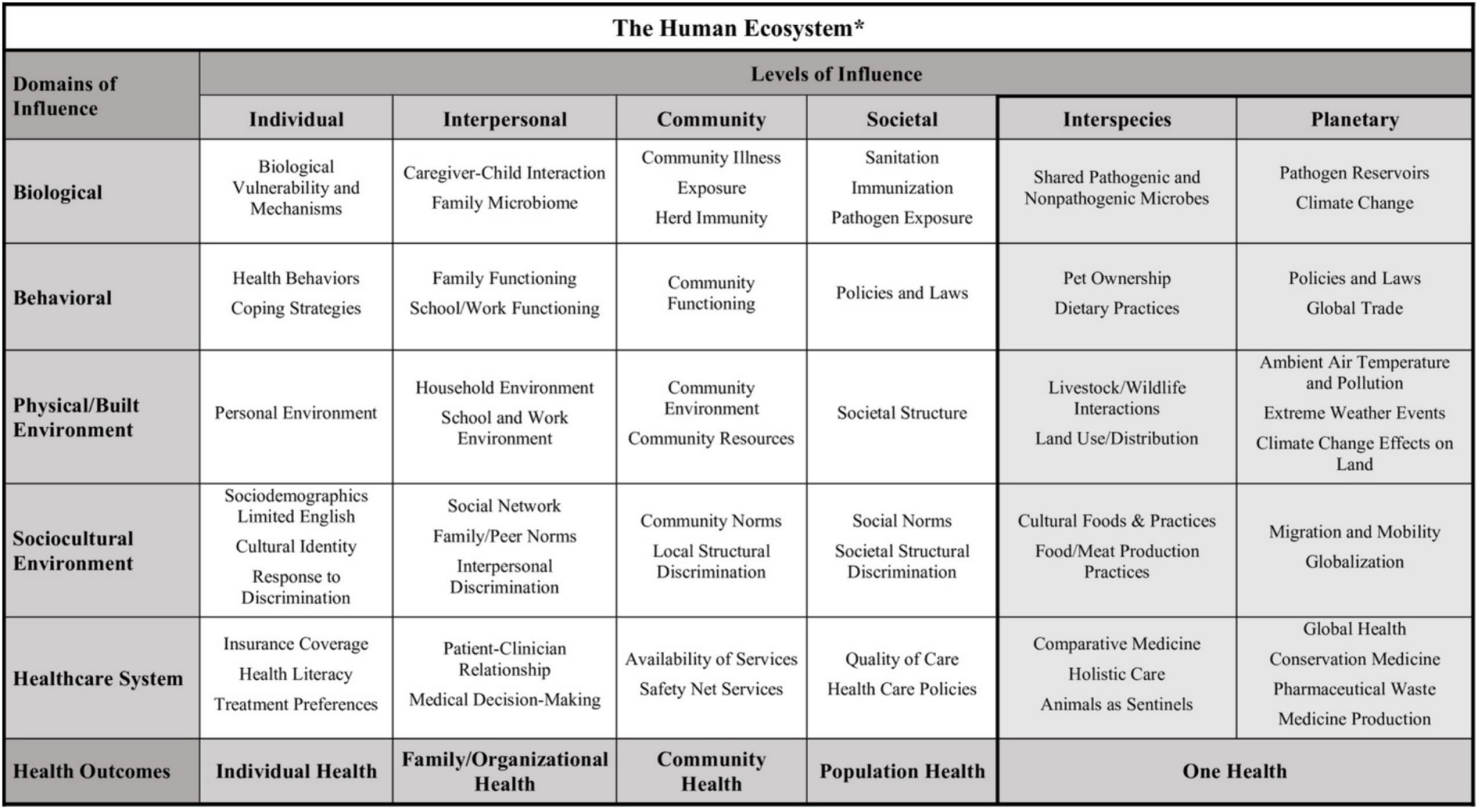
NIMHD framework with One Health addition. Figure drawn from Morgan et al. (36). Canonical URL https://creativecommons.org/licenses/by/4.0/.

Morgan et al. (36) proposed the integration of a “One Health” approach to the NIMHD framework, adding interspecies and planetary levels of influence to capture some of the most salient arenas in which our interdependence with natural systems materializes (Figure 1). Their stated goal was to reflect “how human health is a product of the human ecosystem, which combines traditionally recognized ecosystem components (plants, animals, microbes, physical environmental complex) with the built environment and social characteristics, structures, and interactions between all these elements” [(36) p. 3].

The first author conducted data analysis with a code book using the levels from the One Health addition to the NIMHD framework (individual, interpersonal, community, societal, interspecies, and planetary) as parent codes, each with sub-codes replicating the framework’s domains (biological, behavioral, physical/built environment, socio-cultural environment, and healthcare system). Following data analysis, the authors met via Zoom to discuss the analysis and co-interpret their significance within the team’s lived experiences and knowledge of the literature.

### 2.5 Ethical statement

This research was approved by the University [anonymized] internal review board (protocol #2010166335). To protect the privacy of immigrant participants, we utilized a verbal consent process and reminded participants of the voluntary nature of project participation. Research was conducted in the language and location of participants’ choice, and immigrant participants were given $50 gift cards for their participation. All quotes in this article were translated by the first author who is a trained translator and then verified by the second author, a native Spanish speaker.

## 3 Results

This section presents the integrated results from interviews with both samples. The first sample included ethnohistorians of southern Arizona history, rural landholders and long-time residents, and environmental educators. Five of the seven expert interviewees also had lived experience as Mexican-origin residents of southern Arizona rural environments.

The second sample was composed of Mexican immigrants working in land-based occupations (Table 1) and included eight men and two women. Participants worked in various land-based fields and eight of them were living in Tucson, Arizona and two on rural ranches. Participants were evenly split between having been raised in rural versus urban environments in Mexico before migrating to the U.S.

**Table 1:** Immigrant sample demographics.

	*n* = 10	
	No	%
Current age		
35–45	5	50
46–55	1	10
56–65	4	40
Age at migration		
1–20	4	40
21–40	5	50
40–60	1	10
Gender		
Male	8	80
Female	2	20
Geographic origin in Mexico		
Rural	5	50
Urban	5	50
Where live in US		
Tucson	8	80
Nogales	1	10
Douglas	1	10
Land based occupation		
Community Gardener	3	30
Cowboy	2	20
Mycologist	1	10
Environmental Activist	2	20
Rainwater Harvesting	1	10
Arborist	1	10
Binationally Mobile (yes)*	7	70

**Figure 2:**
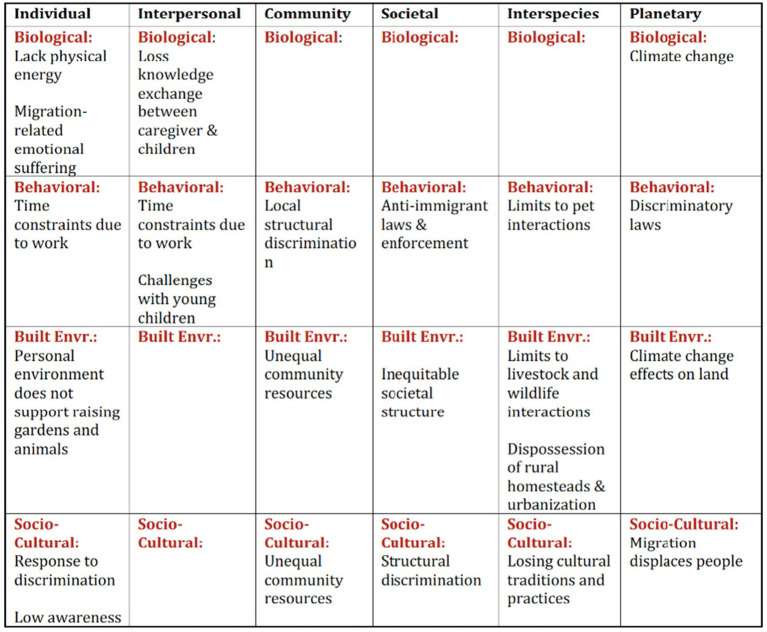
Nature barriers identified by study sample across levels and domains of influence.

We present the study results about nature barriers faced by Mexicans in southern Arizona according to the levels of influence outlined in Morgan et al.'s (36) One Health adaptation of the NIMHD research framework, beginning with the outermost, distal level (planetary) and concluding with the innermost level (individual). Barriers were identified along all six levels of influence and across four of the five possible domains of influence (none were noted in the healthcare system domain) (Figure 2).

### 3.1 Planetary level

As the most upstream level of influence, barriers observed at the planetary level included factors that had broad reaching impacts on human experience irrespective of or across geopolitical boundary lines. The barriers to nature access observed at this level had far-reaching impacts on participants’ lives and were highly intersectional with the barriers that participants reported in other levels of influence.

#### 3.1.1 Climate change and its effects on the land (biological and physical/built environment)

Participants commonly referred to the impact of climate change on the land, noting that the natural spaces that they felt connected to in early life had undergone aridification, limiting the ways that they and others could engage with the land.

Historical experts and stakeholders described the broad desertification of the physical landscape of southern Arizona, which had at one time supported productive small-scale farms and ranches owned by Mexican families. The desiccation of the region was posited to have contributed to the decline in Mexicans’ rural, land-based lifestyles that dominated the region up until the early-20th century. An ethnohistorian explained: “And, of course, the drought had a lot to do with what happened. That was very devastating for *Mexicano* ranchers.” Another historical expert who had lived experience growing up on a ranch recalled: “I was born in ‘32, and the San Pedro River was always running back then. There were motorboats to cross from one side to the other.” She observed that in the wake of diminished access to water and pressure to sell off small land holdings, “there is nothing there now! There is no more ranch, there’s nothing left there!”

Others noted that the drying of the rivers and the landscape reduced opportunities for rural livelihoods and recreational experiences for families and contributed to a decline of intergenerational knowledge exchange around nature on both sides of the border. One local stakeholder in barrio sustainability recalled how time spent walking along a now desiccated river near his ancestral home in Oaxaca had taught him culturally-rooted lessons about how natural, spiritual, and human forces are linked. He recalled: “Like, my family, we used to go to, like, the river and we would talk about the river and that’s where me and my brothers and sister, we learned about the *nahuales*, which is like, not a spirit animal, but kind of, like, a spirit animal in, like, the traditions of Oaxaca.”

#### 3.1.2 Migration displaces people from ancestral lands (physical/built environment and sociocultural)

Participants noted that migration displaced people from their ancestral homelands and frustrated their ability to reconnect with the natural spaces and the land-based practices of their early lives. Oftentimes, these displacements from rural homes to urban locales had occurred pre-migration, reinforcing that planetary level factors defy geopolitical boundaries and often share a common genesis. One local stakeholder described his conversations with Latino immigrants in the US: “and I’m like ‘what brings you here?’ They’re like, “work.” ‘So, what did you do before?’ ‘I used to, like, farm.’ ‘And why cannot you?’ And like, the general story, right, the average story, is ‘oh, we could not sustain our land’. ‘We had to sell our land’ or ‘our land was taken away, so we had to move and find work.’“.

Both domestic and international migration was associated with a loss of rural ties and the adoption of urban lifestyles in which land-based activities were not prominently figured. An immigrant participant who works in rainwater harvesting installation stated that: “in an urban city usually a river like that, a river with running water, it is usually really far away to get to a natural river.” He recalled how in his rural home in the central Mexican state of Jalisco: “for us [the river] was so close by, I think it was about a block away from where we lived.” Other participants noted that immigrants felt uprooted in urban environments where they had no land to tend and commonly expressed nostalgia for specific plants, flavors, and aromas.

Migration was also associated with Mexicans becoming “stuck in one place,” unable to reconnect with their ancestral homeland and the natural spaces and products that sustained them in their youth. An immigrant participant noted that “there’s thousands of people from this side, who do not go to the other side for whatever reason and hundreds, if not millions, of people from that side, they cannot come this way.” This stuck feeling was due in part to the geographical distance from their places of origin and economic constraints that prohibited frequent travel, especially among large families.

But participants also highlighted the impermeability of the geopolitical border between the US and Mexico as constricting immigrants’ freedom of movement. While this was especially the case for undocumented immigrants, an immigrant participant with a valid visa for crossing the border explained that the international boundary line deterred movement across the land for others as well. He stated: “when I was going back to Nogales, there was some sort of red flag on my visa or something. That was super annoying, I was like, ‘I’m so tired of dealing with... immigration, so annoying, and I cannot go’... So, I feel like I do not have the freedom, it’s kind of constricting a little bit, with immigration.”

### 3.2 Inter-species level

Barriers observed by participants at the inter-species level hindered immigrants’ ability to engage directly with flora and fauna due to restrictions in their home environments as well as historical dispossession of Mexicans’ land in southern Arizona.

#### 3.2.1 Limits to livestock and pet interactions (behavioral and physical/built environment)

Many immigrant participants mentioned that they wanted to raise chickens or other small, domesticated animals but were unable to do so due to constraints of living in apartments, small urban homes, or trailer parks. An immigrant participant stated: “I would just have to move to a big place with more land. And I would definitely get chickens.” In addition to limited physical space, other barriers included close proximity to neighbors who might be irritated by animal noises and smells, renting rather than owning their property, and restrictive housing regulations. An immigrant participant stated not owning animals because: “we lived in a trailer... and they did not let us. They are so close together.”

The ability to grow foodstuffs and other plants was also restricted by limited space and the lack of long-term connections to the property. One immigrant participant explained: “I am inside the trailer park, I mean, the land does not belong to me. So, there is always that limitation of, if you grow, like why would you grow so much if it will all stay behind”? A local stakeholder described a deep disconnection from the land more broadly due to being forced to frequently leave family homes due to financial constraints. He recalled: “when we drive around the neighborhood, we are like ‘look at everything that is around us, everything that is around us is meant to cage us in, it’s meant to hurt us, to kill us sooner or to exploit us, and nothing really connects back to the land.’”

#### 3.2.2 Dispossession of rural homesteads and urbanization (physical/built environment)

Historical changes to land-use distribution in southern Arizona that limited interactions between Mexican origin residents and flora and fauna were observed in the physical and built environment domain. Historical experts and stakeholders recalled how Mexicans’ homesteads were lost during extended periods of land fraud and land speculation from the Mexican American War through the mid 20th century, a result of discriminatory practices also noted in the societal level of influence. In addition, participants noted that Mexican families gradually moved toward the cities for educational opportunities, modern comforts, and upward social mobility, leaving families dispersed and causing a decline in land-based livelihoods and traditions of outdoor family gatherings.

One elder participant recalled: “We were out there playing a lot since we grew up at the ranch... So, it was going out into the fields, and we went out into the desert and a lot of it was, a lot of our childhood was spent outside, so to me that was perfect. Unfortunately, I could not really provide that for my children in the sense of where we ended up living. We ended up living in the town, you have to be more careful.”

Participants observed that these historical trends had the effect of limiting opportunities for rural livelihoods among subsequent generations of Mexican origin residents, making it harder to establish themselves in rural regions and to move freely across the land and engage with its plants and creatures. One historical expert recalled that during her childhood:

“[There were] so many places that we used to be able to go, no one had any problem with us. We would go cut wood for our fireplaces and the ranchers would appreciate you cutting down the mesquite and hauling it out of there... my nana would go and gather plants and stuff, nobody cared that we were on their property, and we were respectful... now everything is ‘no trespassing’, the gates are locked, ‘do not touch my property’.”

#### 3.2.3 Losing cultural traditions and practices around land and food (sociocultural)

Participants also noted that the myriad nature barriers that immigrants faced contributed to a decline in culturally rooted traditions, knowledge, and practices related to how humans utilize and engage with plants and animals for food, medicine, shelter, and spiritual connection. This decline was understood as a self-perpetuating cycle that in turn discouraged further engagement. A local stakeholder explained how ecological knowledge was in its essence tied to the land: “I mean, you know, it’s sad when we have to leave our land, you know, and the only way that we find that connection, that remembrance, is by taking care of it, tending it, building that relationship to it no matter where we are at.”

Participants linked these declines in oral tradition and knowledge to both the international migration process as well as urbanization and the loss of rural livelihoods in Mexico. A local stakeholder who was himself an immigrant lamented: “And all the knowledge is just there. Nothing can be practiced because they are not in the fields anymore and yet, they know that knowledge. And in the family, the kids, the grandkids, do not appreciate that... So that is where that knowledge is all over the place and unfortunately is disappearing, more so in Mexico, nowadays.”

Moreover, the increasing impermeability of the international border and the declining rural lifeways in Arizona interrupted the long-standing shared agrarian culture of the region that was embedded in land-based knowledge, family tradition, and spirituality. A rancher who had longstanding relationships with Mexican cowboys and cattle and horse vendors noted that there is less cross border exchange of labor, knowledge, and animals now, which he attributed to young men in rural and urban locales being brought into drug sale and production. Another historical expert participant explained:

“And I would say for at least the first century of its existence, [Arizona] was a northern finger of Sonora. Families with ties, the same way of raising livestock, the same way of farming and irrigating from the Santa Cruz River... The same fiestas in many cases, the San Isidro, the patron saint of farmers, El Dia de San Isidro is still celebrated in agricultural communities in Sonora, and it was celebrated in Tucson until at least the early 20th century. And the border was really very porous at least into the 1920s, so I think for many people there was this feeling of familiarity based upon the landscape itself because many of them came from the Sonoran Desert or they came from grasslands in Sonora to Southeastern Arizona and also the family ties, which were in some cases six seven generations strong.”

Participants also noted that the loss of connection with both plant and animal food sources had detrimental effect on nutrition and holistic food traditions rooted in wild-harvesting, growing one’s own food, and making meals from scratch. An immigrant participant who worked as a mycologist commented that it was an issue of: “what they have access to really. Even some of the people here, they do not have a lot of knowledge about the cacti they can eat, so, and the fruits of the cacti, the plants.”

### 3.3 Societal level

Factors observed at the societal level reflected the impact of state and federal legislation and institutionalized power structures that have curtailed and restricted Mexicans’ access to live and work in natural spaces. The impact of these societal level factors reverberated both outward to the One Health levels of influence and inward toward the community, interpersonal, and individual spheres.

#### 3.3.1 Anti-immigrant policies, laws, and enforcement (behavioral and sociocultural)

Participants frequently cited state immigration laws and local enforcement of these laws as limiting immigrants’ freedom of movement, both within Arizona and binationally. Because these laws were considered to discriminate against Mexican origin people, they are listed in both the behavioral (laws) and sociocultural (structural discrimination) domains.

Participants noted that immigrants’ access to outdoor spaces in and around Tucson was limited by the enforcement of anti-immigrant legislation, particularly State Bill 1,070 which went into effect in 2010 and allowed police to inquire about immigration status and to communicate directly with U.S. Immigration and Customs Enforcement (ICE). One stakeholder posed the question: “how can folks, like, go to the Desert Museum without having the fear of being pulled over? Cause that’s a really long trek, right? So, we always have this fear of being pulled over, especially since our state has SB1070 so you can be pulled over and asked for papers at any moment. I think there is like this fear.”

An immigrant participant compared his own freedom to those of his undocumented friends and community members, saying: “They’re very restricted. Really, it’s like they are trapped. They cannot go, they do not have the freedom like I do and like ‘oh I love going here, I love going there’.” Another Arizona law that restricted immigrants’ freedom to access natural and wild spaces was legislation requiring proof of visa or citizenship status to obtain an Arizona driver’s license. An immigrant participant explained how her lack of driver’s license discouraged her family from exploring parks and mountain ranges further afield from Tucson: “And then driving without a license... you really cannot risk it. It is a risk to drive without a license, if something happens and then you know how it is now with the SB1070 law that gives the police the role of immigration authorities. So why put yourself at risk?”

Participants also observed that federal immigration policies and increased border militarization had limited the ability of cowboys and ranch hands from Mexico to cross the border for seasonal work like fixing fences and working cattle roundups. A stakeholder who owns a ranch near the border lamented how long it took his cowboy to cross the border from Agua Prieta, Sonora for work in the morning, recalling “I think it used to be pretty hospitable. I think there was a lot of looking the other way as far as people getting in and then, eventually, they were good, they stay here, they get a green card maybe eventually become citizens. Um, you rarely had border patrol on ranches although occasionally you would.”

#### 3.3.2 Structural discrimination against Mexican-origin people (physical/built environment and sociocultural)

Historical expert participants described discriminatory legal and social practices in deep historical time that put Mexican-origin residents at a structural disadvantage vis-a-vis land ownership and relegated them to poorly paid labor that reduced access to and time for recreational opportunities, as observed in the following two levels of influence.

Several participants recounted from both personal and academic vantage points the history of dispossession of vast and fertile swaths of land from Mexican families along the Santa Cruz River, Altar Valley, and other prime growing regions, following US acquisition of southern Arizona territory in the mid-1800s. One local ethnohistorian whose academic career focused heavily on land tenure in the region said that southern Arizona witnessed: “basically about 140 years of unbroken land fraud in that region, where first the O’odham community of Tumacacori and later predominately Mexican settlers homesteaders along the Santa Cruz River were just disposed, forced off their land, primarily by speculators.”

Historical experts also noted that under U.S. control, Mexicans in the region were the victims of discriminatory housing practices, unequal wages, and overt discrimination that contributed to their declining social, political, and economic standing that have lasting impacts to the present-day receiving environments for Mexican immigrants. The same historical expert went on to explain:

“Mexicans could not become railroad engineers on Southern Pacific. Mexicans were paid a lower wage for the same work in copper mines and smelters. The dual wage system was instituted in nearly all the mining districts in Arizona and some mining districts were known as “White man’s camps,” where *Mexicanos* were discouraged from settling there or working there, so there was very definitely both legal and informal segregation and discrimination, including in housing.”

### 3.4 Community level

#### 3.4.1 Inequitable nature resources (behavioral, physical/built environment and sociocultural)

Participants observed that at the community level, local structural discrimination contributed to an inequitable distribution of and access to natural resources, which limited Mexicans’ ability to enjoy their natural environment. At the most basic level, the racial profiling that resulted from discriminatory immigration laws vastly reduced Mexicans’ mobility and their perceptions of safety when outside the house. An immigrant participant who works at a local community farm explained how she worried about walking in Tucson, “because they told me not to walk, because here in the United States no one is out walking in the street, so it would look like you do not know how to drive or they are, they do not have documents. And I was like, really? And if they pick you up? And yes, they will even drag you off the bus.”

Participants noted that there was low representation of people of color on local boards and organizations that made decisions about land use, leading to inequitable distribution of city parks, safe paths for walking and biking, and open spaces. One immigrant participant noted a significant difference between the campsites and parks that covered entrance fees versus those that were free and therefore more accessible for Mexican immigrants and other low-income residents. She said, “Yes, I have seen that there also is racism at the camping sites, because there are places where they do not charge or anything, but they do not have, you almost, you almost need to go with a *machete* to clear your own space.”

Several participants described barriers to Mexicans’ ability to enjoy and enhance their urban yards, such as a lack of accessible information in Spanish about what was allowed in residential spaces in terms of small livestock ownership and property amendments for rainwater collection. A historical expert who works in empowering immigrant families at the neighborhood level noted: “it’s hard to know where to find information about what you can do in your own land and about vacant lots and the options for those spaces. I think systemically there’s a lot of barriers, misinformation, not enough information, no transparency as far as what you can do with your own space.”

Others observed that the high concentration of environmental pollutants in immigrant-dense neighborhoods also frustrated full utilization of home gardens and yards. A local ethnohistorian described her childhood home: “It was that little part of land was going to give you it’s reward. Whether it was peaches, or lemons or grapefruit, watermelons and squash. And even in our little plot, that little, small plot that we had on Missouri Street on the South side, that place was a little paradise... Wherever you were, you needed to make it your space.” However, in 1982 her neighborhood was designated as a Superfund site due to high levels of carcinogens in the local water supply from TCE (trichloroethylene) contamination that was linked to elevated rates of lymphoma, leukemia and multiple myeloma along a five-mile stretch. She recalled, through tears, how her mother died prematurely from cancer, being unable to meet her dream to:

“move to a place where she would have more land and have her own space and not have to look off into the distance and would not have another house next to her. I think she always felt that to her that was her dream. So, everything gets so tied into because I do not know if you heard about the TCE problem with the water in the South side. The water was literally being polluted by Hughes [Aircraft] and Raytheon [Missile Systems Co.] and it was on the South side so there were huge canister clusters. And we believe that’s what sickened my mother, and to the point where I have auto-immune problems and so does my daughter so it’s so interesting how life takes you to different places. And I am getting emotional.”

### 3.5 Interpersonal level

The barriers observed at the interpersonal level demonstrate how the broad processes described at the prior levels of influence come to impact intimate human interactions with other people, work and home environments, and with time itself.

#### 3.5.1 Loss of intergenerational knowledge exchange (biological)

Several participants noted a breakdown in intergenerational knowledge exchange about natural systems due to changing dynamics within the caregiver-child dyad and the family dynamic. The planetary phenomenon of migration and the effects of societal laws regulating border militarization were experienced as the separation of children, parents, and grandparents. A historical expert from a binational family observed a break-down in knowledge transmission about respecting and honoring land-based relationships “because of the border and political, political issues, like some of our families cannot even cross to even meet their grandparents.”

Other historical expert participants who were raised in rural areas described how female caregivers used to spend significant time with children on the homestead, during which experiential nature-based learning took place. An ethnohistorian described: “[my mother] was at home, but she was doing all the work of keeping everything together. But we had the time to spend with her, spend the time with her in the garden, to learn about plants, to just sit down and have a cup of coffee outside and enjoy the weather, the shade. And I think that’s what’s been lost. The time to spend, you know, with the children.”

#### 3.5.2 Lack of time to observe and immerse oneself in nature (behavioral)

For many participants, the demands of low-paying work reduced or eliminated the free time necessary for outdoor exploration and for being in tune with natural cycles via tending their garden. One historical expert described how her body yearned for the connection to the harvest seasons that she felt as a child, saying “I miss those times, and I miss scratching in the dirt and starting something and knowing that in a few months I’m going to have *calabacitas*. There’s just not the time and I mourn my loss of that.”

Immigrant participants described feeling constant pressure to earn money and be successful economically which led to a prioritization of work over outdoor leisure time. This pressure combined with the struggle to balance family demands and community-based activities, making nature-based activities logistically challenging. An immigrant participant said: “It’s hard when you work, [you have] the house, and another volunteer organization where I work is [anonymized organization]. And so, it’s so much work, and so I did not have any time leftover to go to my plot at [the community garden].”

#### 3.5.3 Taking small children into nature is challenging (behavioral)

Participants also found that having small children complicated spending time in natural spaces. Some participants noted that children were dependent on cell phones and other forms of screen time and resisted leaving them behind for nature excursions and complained while out in the natural world. One immigrant mother of three children recalled: “the times that we have gone [the kids] have made it really tough but I think that was because they were younger. But now that they are older, that say ‘oh, mami, it’s so close by!’ And I tell them, ‘Remember how you guys always complained?’ And that’s why I do not want to go, because they are always complaining.” Others indicated that it was coordinating multiple busy family members that was the primary challenge, highlighting once again the role that time constraints play in limiting time in nature.

### 3.6 Individual level

Barriers generated in the previous spheres of influence were embodied at the individual level in ways that affected immigrant’s biology and their knowledge and awareness. Two factors observed at this level were previously discussed in prior sections and are not repeated here (lack of land for growing and raising animals and the prioritization of work over outdoor leisure).

#### 3.6.1 A lack of physical energy for outdoor activities (biological and sociocultural)

Many immigrant participants stated that physical exhaustion from working multiple jobs or positions requiring manual labor led them to favor past times that did not involve additional expenditure of physical energy, discouraging them from spending time in nature. This barrier was closely linked to societal level discrimination.

One participant remarked: “The routine is the barrier. Like the routine that I have adopted, for example when I was working as a dishwasher, because after the dishwashing I would go [clean] rugs, and so I was working all day. I would arrive at night... and I would go to bed and fall asleep and not wake up until the next day.” Another immigrant participant described how her work routine consumed all her physical energy: “before when I used to work in restaurants, in a restaurant, and I worked from 8a.m. until 11 at night. So, I worked all week, and I was also so tired and so we did not do anything. And there went 2 years and I did not even realize it.”

#### 3.6.2 Migration-related emotional suffering reduced capacity (biological and sociocultural)

Participants also observed that immigrant’s embodied emotional distress, primarily fear, sadness, anxiety, and loneliness, decreased their interest in and ability to explore their natural environments. These emotions can be understood to be a form of biological vulnerability that is a response to discrimination.

Fear and anxiety were linked to the enforcement of the immigration laws observed in the societal level and acted to reduce immigrants’ comfort level for gathering with others, going to new places, and doing outdoor activities. An immigrant participant who educates immigrants about environmental sustainability described how she felt when she was out in public: “It’s like that pressure that you feel when you walk in the street and the police come up behind you, and your heart starts to pound and I would say to myself ‘this is going to give me a [heart] attack.’”

Others described that the experience of migration resulted in deep isolation, loneliness, and sadness for many immigrants, which could dampen their interest in activities. Oftentimes, these emotions were linked to their lack of freedom to explore, move about, and engage with their surroundings as they had done pre-migration. For others, the negative emotions were more specifically tied to being dislocated from family and culture. Some participants linked these negative emotions to poor health decision-making that favored unhealthy comforts over physical activity. One immigrant participant described: “It’s hard for some people, because well, their family is over there [in Mexico]. All their kids are there. But he is here, he is alone, the depression starts, the alcohol starts, cigarettes, all the vices, and so it gets converted into a vicious cycle which you cannot get out of sometimes.”

#### 3.6.3 Immigrants lack awareness about availability and safety of outdoor resources (sociocultural)

Participants noted that immigrants lacked awareness of opportunities for safe and accessible outdoor engagement in their neighborhoods and community and in Arizona more broadly. In some cases, this was just an issue of being unaware of the existence of outdoor venues. An immigrant participant who works at a local community garden said: “Because they either, do not know, they do not know that this garden is here. And they do not know that we are growing these plants that were [from] their childhood.”

But more commonly the lack of knowledge was tied to fear and distrust surrounding local immigration enforcement. One immigrant participant explained: “I think the first think to tell them is that they should not be scared, right? Tell them to enjoy the place where they live and explore their neighborhoods, because there are many places right here in their own neighborhoods.”

This intersection of societal level anti-immigration laws and discriminatory practices with individual immigrant’s awareness of accessible outdoor opportunities was observed most acutely with recreational resources outside of Tucson, where participants were less familiar with the roads, laws, and social environments. An immigrant participant explained how her son had begged to go camping and how for years she told him to just play at home. She said: “I want to go camp at the Grand Canyon and take my son, but yeah, what I have realized is that you have to walk a lot, or things like that, that there are certain limitations. But since I have not been there yet or gone with anyone else, I have not seen the route. So, I just do not feel that safety to go, because I do not know how.”

Finally, a local stakeholder added that the lack of culturally relevant outdoor education models excluded immigrants from mainstream outdoor education models and outreach efforts. He explained: “When I started doing this work, there wasn’t really any anything written or anything that I could use to teach you know, Barrio Sustainability or Barrio Campesino style... so I had to create a lot of that.”

## 4 Discussion

This article responds to the NIMHD’s call for a more intersectional approach to minority health by highlighting the intersectionality of place, migration status, socioeconomic factors, and discrimination in shaping immigrants’ access to nature (30, 37). In addition, our incorporation of the One Health adaptation of the NIMHD framework for data analysis evidenced the deleterious impacts of upstream barriers from the planetary and societal levels upon immigrants’ daily engagement with other species, the natural environment, family and community, and culturally grounded ecological learning.

Our ethnographic approach to exploring immigrant’s lived experience of barriers to nature via the migration process highlighted their “critical consciousness” (23, 38) of how discriminatory policies and marginalizing historical forces shaped their access to and relationship with the natural environmental. They described facing barriers to nature access that have been previously documented among Latino immigrants, including limited awareness of outdoor resources, lack of time and energy due to work demands, fear of detention or deportation due to undocumented immigration status, socioeconomic constraints, and the poor condition of local natural resources (3, 4, 5, 6, 25, 39). But participants also highlighted barriers that received less attention in the literature including the migration process itself, climate change, emotional suffering, a breakdown in intergenerational knowledge exchange, and housing regulations that limit interspecies engagement.

Migration, which originated on the planetary level, had the initial impact of displacing people from land that they were intimately connected to. This subsequently set the stage for the processes of urbanization and the dispossession of Mexicans’ rural homesteads that reduced time spent in nature and separated Mexicans from the plants, land, and animals they had once tended. This phenomenon of displacement has been widely observed and documented throughout the US southwest (40–42), leading to what which Zentella (19) calls “the loss of identity as a landed people” or “losing the mother” (p. 187). Participants described how displacement ultimately culminated in the breakdown of ecological practices that previously had supported land-based knowledge exchanges among family members (43). It also produced the biological manifestation of sadness and loneliness that further dampened immigrants’ will to engage with their natural environment (5).

The migration process also placed immigrants under the jurisdiction of a new society, where anti-immigrant laws observed on the societal level curtailed their freedom of movement across the land, an impact embodied on the individual level as fear and anxiety connected to exploration of their surroundings (24, 44). Discriminatory practices observed on the societal level likewise relegated immigrants to working physically demanding jobs (45) that disrupted biological patterns of ecological knowledge exchange between caregivers and children and depleted their time and energy for nature exploration. Existing research has shown that a majority of immigrant workers cite that low pay and pressure to work and earn money disrupts their prior “rhythm” of life and reduces time and energy for family connection and leisure activities (46). Moreover, we observed how participants’ low socio-economic status reduced their inter-species interactions via housing regulations prohibiting domesticated animals in the trailer parks and apartment complexes where they could afford to live, as well as via housing instability which reduced their investment in and ties to land.

In addition, climate change occurring at the planetary level altered landscapes on both sides of the border, intensified urbanization and displacement and undermined streams of ecological and place-based knowledge and practices that previously bound immigrants to their environment (47). This finding is supported by significant evidence that climate change inequitably harms low-income, Indigenous, and other vulnerable populations that are more dependent on the land and thus are more directly exposed to the damaging effects of global warming (48, 49).

### 4.1 Applied implications

A more concerted effort is necessary to increase nature access among Mexican immigrants in the US. Nonetheless, less than 7% of studies exploring the health benefits of nature include interventions to promote tangible gains in nature access, reflecting a broader fragmentation of our approaches to the study of human health and the environment (21, 50). This is a missed opportunity to buffer against health risks for immigrants, who may face increased potential health benefits from re-emplacement in their new receiving areas (4).

Promoting health via increasing nature access can take diverse forms, including visiting urban parks and community gardens, being immersed in “wild spaces,” or even *watching* nature from inside a car or a hospital room (51–53). Efforts to increase land engagement and related resilience among immigrants and other displaced populations should center culturally-grounded projects such as ancestral land and farming initiatives and employ community driven outreach methods that highlight the whole family and incorporate community health worker and other vetted models (61). Land-based projects centering growing food and promoting healthy eating may work to revive pre-migration land-based knowledge and ecological relationships to food production and preparation (54). Community gardens that integrate cultural and ethnic heritage in their programming and recruitment efforts have been shown to expand cultural expression and increase cultivated biodiversity (55, 56) as well as to offer displaced communities a cherished space for “home-making” in relationship to the natural elements of plants, animals, water, and earth (57). Health intervention studies that work to facilitate immigrants’ access to and participation in such culturally grounded programming can expand the field by measuring their impact on reducing barriers to nature via specific, evidenced-based strategies as well as the associated human health benefits of this expanded access and impacts to immigrants’ interest in and success as environmental stewards.

### 4.2 Strengths and limitations

In this article we utilize a deeply contextual, place-based, and nuanced anthropological lens to expand upon the diversity and intersectionality of barriers to nature access among Mexican immigrants. However, study results are limited by a small sample, and by the fact that the immigrants we interviewed had successfully navigated their way to nature-based jobs and volunteer positions, which may limit their full understanding of barriers faced by other immigrants.

## 5 Conclusion

Morgan et al. (36) affirm that nature barriers have an impact on the wider network of the human-animal-ecosystem, and evidence shows people who spend more time in nature make better nature stewards (58). Blazing a path forward in which all species gain from respectful and mutual interactions necessitates the full engagement and inclusion of Mexican immigrants, Indigenous groups, women, and others who have lacked a seat at the decision-making table and whose environmental stewardship and embedded worldviews offer integrated and bidirectional solutions for optimizing human-nature relationships (19, 59, 60).

## Data Availability

The datasets presented in this article are not readily available because the participant sample included immigrants who preferred not to have their data shared to protect their identity. Requests to access the datasets should be directed to rcrocker@arizona.edu.

## Footnotes

^1^ Dedoose.com

## Appendix

**Table A1:** Relevant interview questions from immigrant interview guide.

Focus area	Sample questions
Relationship to land	“Are you able to cross back and forth between Mexico and the US? If yes, how frequently do you cross?”
	“Would you say that you ever feel lonely for the place where you were raised? What parts of it specifically (smells, views, rain, etc.)”
	“Are there things that make living and working in southern Arizona difficult? If yes, what?”
	“In what ways is your relationship to your home here different than it was in Mexico (i.e., owned versus renting, was it ejido ownership, had more land, intergenerational, etc.?)”
Barriers to land engagement	“Does the physical environment in Arizona limit your ability to raise animals/garden/engage in outdoor recreation? (i.e., excessive heat, frost, lack of water)”
	“Does the political and social environment limit your ability to raise animals/garden/engage in outdoor recreation? (ie. permitting, zoning codes, noise complaints, fear of detection, public health codes).”
	“Do economic constraints limit your ability to raise animals/garden/engage in outdoor recreation? (ie. lack of land, hard to buy feed, pay for water, permits, etc.)”
	“Do you think it’s important for other Mexicans in southern Arizona to have increased access to natural spaces and the outdoors? If yes, why?”
	“What are the primary barriers that prevent other people in your community from getting outside and spending time in nature?”
	“What do you suggest we/you could do as a community to reduce those barriers?”
	“Are you satisfied with the amount of time you personally spend outdoors and connected to nature? If not, what would you need to be able to do it more?”
